# Assessing neural responses towards objectified human targets and objects to identify processes of sexual objectification that go beyond the metaphor

**DOI:** 10.1038/s41598-019-42928-x

**Published:** 2019-04-30

**Authors:** Jeroen Vaes, Giulia Cristoforetti, Daniela Ruzzante, Carlotta Cogoni, Veronica Mazza

**Affiliations:** 1 Department of Psychology and Cognitive Sciences, University of Trento, Trento, Italy; 2 Center for Mind/Brain Sciences, University of Trento, Trento, Italy; 3 Department of Experimental Psychology, University of Ghent, Ghent, Belgium

**Keywords:** Social neuroscience, Social behaviour

## Abstract

Objectification – reducing a someone to a something – represents a powerful and potentially damaging way in which we can see and treat others. Women are often victims of processes of objectification that occur whenever a woman is reduced to her body or certain body parts. What remains unclear is the extent to which a woman becomes an object when objectified. Using the oddball paradigm in three experiments, participants’ neural activity was measured while they analyzed frequently presented male and female human stimuli and infrequently presented gender-matched doll-like objects. The infrequent doll-like objects were expected to trigger a late event-related neurophysiological response (P300) the more they were perceived different from the repeated, human stimuli (i.e., the oddball effect). In Experiment 1, the oddball effect was significantly smaller for objectified women compared to objectified men. Results of Experiment 2 confirmed that this effect was confined to objectified depictions of women. In Experiment 3, no semantic references to the human-object divide were provided, but objectified women were still perceived more similar to real objects. Taken together, these results are the first to demonstrate that the perception of women, when objectified, changes in essence beyond the metaphor.

## Introduction

Our interactions among humans are typically determined by our willingness to know other people’s thoughts, attitudes, desires and intentions. Our interactions with objects, instead, are mostly guided by their usefulness and appearance. These typical interaction patterns are normally clearly distinct as separate brain regions subserve the elaboration of human vs. non-human stimuli^1^. Still, there are instances when the human-object divide tends to fade. This occurs when people objectify other human beings. Objectification occurs whenever a someone becomes a something. In the case of sexual objectification, this someone is typically a woman whose body or body parts are seen as mere instruments, separated from her personality and individuality, regarded as if they were capable of representing her^2,3^. Hence, much like objects that are mostly valued for their appearance or usefulness, when objectified, women are especially appraised for their attractiveness and instrumental value. What remains unclear is whether objectified women become truly similar to objects or whether the object reference is a mere metaphor.

Sexual objectification is prevalent in modern Western societies and it mainly targets young women. In a recent Australian study^4^, young women reported to undergo an objectifying event (e.g., unwanted body gaze, catcalling, sexual remarks, groping, and sexual gestures) every other day and witness the sexual objectification of others, both through the media and in interpersonal interactions, approximately more than once on a daily basis. The representation of women in the media is often objectifying and hardly compensated with more empowering imagery in most of the Western world^5,6^. Such direct and indirect objectifying experiences have consequences that negatively impact women’s self-views^4,7,8^ and in the long run potentially jeopardize their well-being^9–12^. Moreover, perceiving a woman in objectifying terms increases sexual harassment^13–15^. Therefore, getting a better understanding of the processes that underlie sexual objectification is of utmost importance.

A unique focus on the female rather than the male body in objectification research has been motivated by both evolutionary and socio-cultural theories. From an evolutionary perspective, the female body attracts more attention compared to the male body because it typically has a cluster of cues that provide information about a woman’s fertility and reproductive value^16,17^. Socio-cultural theories, instead, have emphasized the impact of stereotypical roles^18^ and the patriarchic hierarchy as causes that keep women’s evaluation especially based on their appearance^2,19^. Both theories potentially explain why women are more likely victims of objectification reducing them to their body appearance or to certain body parts. As a result, the female body is more likely valued for its appearance and its usefulness, much like an object.

The change from someone to something has been probed in research on dehumanization and anthropomorphism; it has been shown that dehumanized outgroup members and (disgusting) objects elicited similar brain patterns^20,21^, while anthropomorphized objects induced similar neural responses compared to human stimuli^22–25^. In the realm of sexual objectification similar research efforts have been conducted but none have allowed us to truly gauge the similarity between objectified women and real objects.

Work on dehumanization^26–29^ has shown the associations, metaphors, or trait attributions that people make when they are confronted with men and women depicted in swim- or underwear (i.e, objectified) or fully-clothed (i.e., non-objectified). Objectified women were described as less capable, mindful, and friendly or were associated more easily with animal terms (e.g., nature, snout) compared to scarcely dressed men and fully-clothed women. While these results give us an idea of the semantic associations that people make when they are confronted with objectified women, they do not allow us to infer that these women actually become more similar to objects at a perceptual level.

In a similar vein, neuroimaging results^30^ revealed that men with hostile sexist attitudes towards women showed decreased activation of those brain areas that are typically associated with mind attribution processes when looking at objectified women compared to other social targets. Other research has suggested that objectified female targets are elaborated using cognitive processes that are typically used in our interactions with objects. While objects are typically recognized using analytical processing, recognition of people and especially human faces is achieved through configural processing. Given that the latter process implies that successful recognition depends on the perception of the relations between the constitutive parts of the stimulus, the recognition of people is typically inhibited when their body or face is inverted, while the recognition of objects remains unaffected (e.g.^31,32^). Applying the inversion effect to the realm of sexual objectification, Bernard *et al*.^33^ found that unlike other human targets, no difference occurred in the recognition of objectified female bodies when they were shown upright or inverted. In other words, objectified female bodies were fragmented and recognized as a recollection of body parts, a piecemeal process that is typically observed in the recognition of objects. Showing that certain brain areas^30^ or cognitive process^33^ are similarly involved when elaborating both objects and objectified women, however, does not guarantee that they are actually the same thing or even become similar. For one because under certain conditions objects have shown to trigger inversion effects as well^31,34,35^ meaning that there is no perfect overlap between the type of process (analytical vs. configural) and the target (object vs. human). Moreover, stimuli that are very different, like tasty food and illegal drugs, are known to activate the same brain regions (i.e., the reward system^36^).

To gauge the true similarities between objectified women and real objects, one should (1) make a direct comparison with objects and (2) use a procedure that directly assesses the perceptual similarities between object and human stimuli, instead of just measuring a similar processing style. Attempts to test the first point have been conducted recently. Focusing on the N170, an event-related potential typically associated with configural processing, research found that only non-objectified (i.e., fully-clothed) human bodies were processed configurally unlike objectified (i.e., scarcely dressed) human bodies and objects (i.e., shoes) when the stimuli were either scrambled^37^ or inverted^38^. Similarly, in another study the inversion effect was observed for non-objectified women, but not for objectified women and objects like houses^34^. While these studies managed to test a similar processing style adopted when recognizing pictures of objectified women and real objects, no attempt has been made to directly test the similarities in the perception of both objectified women and real objects. Therefore, previous research did not allow us to conclude that objectification processes go beyond the metaphor implying that objectified women truly become more object-like. The current studies introduce a novel paradigm that directly compares participants’ neural activity when they are confronted with pictures of (non-)objectified men and women and comparable objects and allows us to measure the true similarities between human and object stimuli.

## The Present Research

To test the hypothesis that objectified women are perceived more similar to objects compared to other human targets, three experiments were conducted. In all experiments, the well-known oddball paradigm was adopted (e.g.^39,40^), in which a sequence of repetitive stimuli is infrequently interrupted by a deviant stimulus, i.e., the oddball. Event-related potentials (ERP) were recorded in an active condition and the response towards the oddball and the repetitive stimuli were analyzed. Research using this paradigm has shown that the P300 – an event related potential component that occurs around 250–600 ms after stimulus onset – is triggered by the infrequent stimulus and its amplitude increases to the extent that the oddball stimulus is perceived as different from the repeated stimuli^41,42^. In Experiment 1, the repeated items were either objectified (i.e., scarcely dressed) male or female targets, while non-objectified (i.e., fully-clothed) female and male targets were repeatedly presented in Experiment 2. In both experiments, the infrequent targets were perceptually comparable objects (i.e., doll-like avatars) that were specifically tailored for the purpose of these studies. According to our hypothesis, the P300 should be significantly smaller when a female doll-like avatar appears among a set of objectified female pictures compared to when a male doll-like avatar is infrequently presented among a series of objectified male pictures. In contrast, we did not expect a similar difference to occur in Experiment 2, given that all the stimuli depicted non-objectified targets. The results of this study allowed us to show that not women in general, but only objectified women are perceived more similar to objects. Finally, in Experiment 3, only objectified targets were presented, but unlike the previous experiments, the categorization task was unrelated to the human-object divide. Eliminating all semantic reference to humans or objects allowed us to further corroborate the hypothesis that the woman object is not a mere metaphor but conveys true similarities with real objects.

## Stimulus Creation and Pre-Test

**Figure 1:**
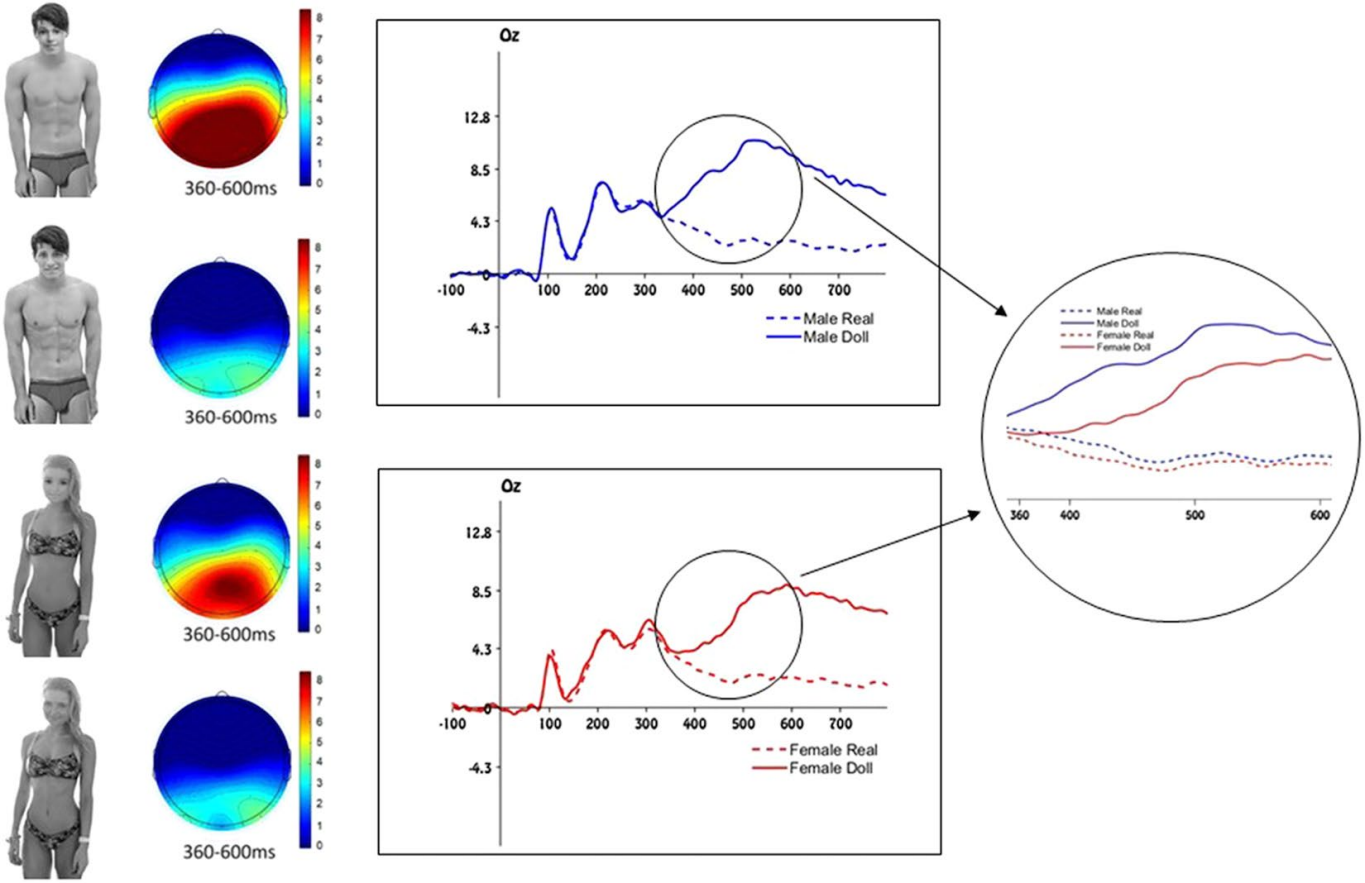
Stimuli and electrophysiological results of Experiment 1. Left panel: example of stimuli depicting an objectified human male, an objectified human female and their respective doll-like avatars. The specific stimuli that are shown in this figure were not used in the current experiment, but are similar to the originals. Due to copyright restrictions we cannot publish the original experimental stimuli. The experimental stimuli can be obtained on request contacting the corresponding author. Mid panel: scalp distribution of the ERP activity in the P300 time window. Right panel: Grand average waveforms for objectified male and female targets and their respective doll-like avatars. Right circle: Detail of the comparison between the grand average waveforms between all targets in the P300 time window.

**Figure 2:**
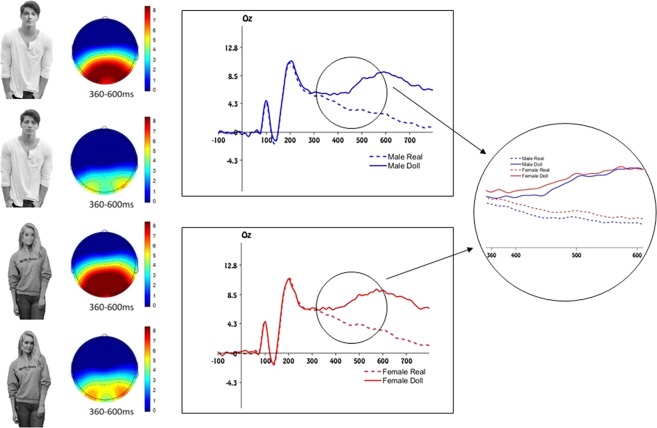
Stimuli and electrophysiological results of Experiment 2. Left panel: example of stimuli depicting a non-objectified human male, a non-objectified human female and their respective doll-like avatars. The specific stimuli that are shown in this figure were not used in the current experiment, but are similar to the originals. Due to copyright restrictions we cannot publish the original experimental stimuli. The experimental stimuli can be obtained on request contacting the corresponding author. Mid panel: scalp distribution of the ERP activity in the P300 time window. Right panel: Grand average waveforms for non-objectified male and female targets and their respective doll-like avatars. Right circle: Detail of the comparison between the grand average waveforms between all targets in the P300 time window.

**Figure 3:**
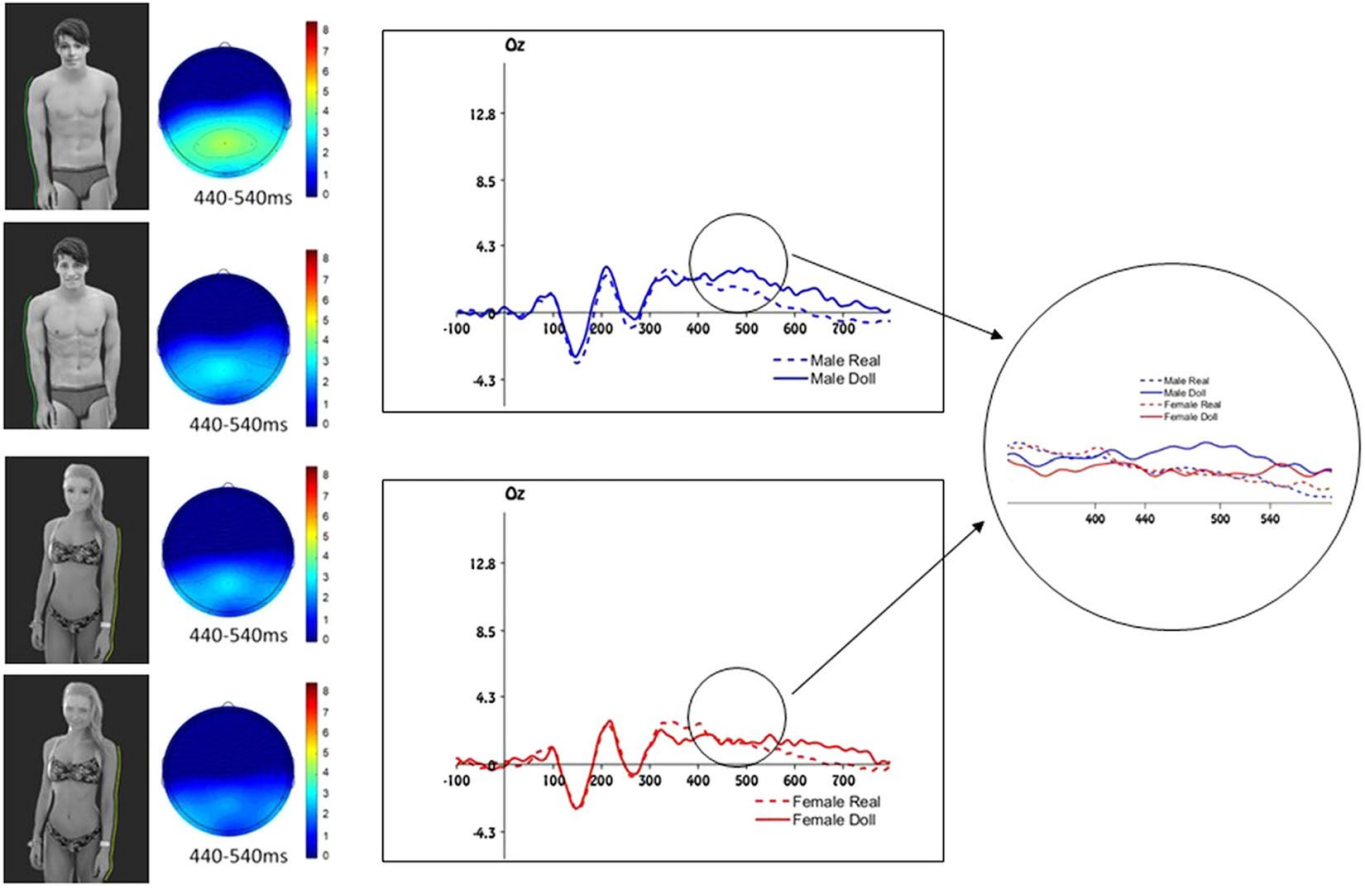
Stimuli and electrophysiological results of Experiment 3. Left panel: example of stimuli depicting an objectified human male, an objectified human female and their respective doll-like avatars. Yellow or green contour lines were applied on the right or left side of each target stimulus. The specific stimuli that are shown in this figure were not used in the current experiment, but are similar to the originals. Due to copyright restrictions we cannot publish the original experimental stimuli. The experimental stimuli can be obtained on request contacting the corresponding author. Mid panel: scalp distribution of the ERP activity in the P300 time window. Right panel: Grand average waveforms for objectified male and female targets and their respective doll-like avatars. Right circle: Detail of the comparison between the grand average waveforms between all targets in the P300 time window.

A total of 82 pictures were selected from websites on the internet. We followed the same assumption as in previous research (e.g.^27,28^) sustaining that men and women who appear in swim- or underwear attract more attention to their body and therefore are more likely objectified. The pictures represented 21 women and 20 men each appearing in swim- or underwear in Experiment 1 and 3, while the same models were fully-clothed in Experiment 2 (see example stimuli in Figures 1, 2 and 3). All models were depicted from the knees up and watched straight into the camera. Models with explicitly sexualized body postures or extreme facial expression were avoided. All pictures were converted to greyscale to equalize their luminance as much as possible. For each picture, a doll-like avatar was obtained creating a morph between the original face of the model (30%) and a doll-face (70%) and applying a surface blur on the visible skin of each model’s body (see example stimuli in Figures 1, 2 and 3). The stimuli were pre-tested through an online questionnaire in which 22 participants (12 female) categorized each picture as either an object or a person. Both the human pictures and the doll-like avatars were correctly recognized as a person or an object respectively (98% correct responses in both cases). Importantly, the recognition accuracy of the pictures did not change as a function of the way they were dressed, the gender of the targets, or the participants’ gender. In the same questionnaire, and for the human pictures only, we asked participants to indicate on a 7-point Likert scale to what extent the picture portrayed an objectified man or woman. In line with previous research^27,28,30^, both male and female targets were judged to be objectified to a greater extent when they were presented in swim- or underwear (*M* = 3.05, *SD* = 0.37) compared to when they were fully-clothed (*M* = 2.25, *SD* = 0.26), *F*(1, 20) = 13.27, *p* = 0.002, *η*^2^_*p*_ = 0.40. Importantly, this effect was not moderated by both target or participants’ gender (see Supporting online Information for a full analysis).

## Experiment 1

In Experiment 1, the oddball paradigm consisted of objectified female and male targets; doll-like avatars reflected the infrequent stimuli that appeared within an array of frequent objectified human stimuli. Participants had to indicate, as accurately and as fast as possible, whether each target portrayed a human or an object-like avatar by means of a key press.

### Results

#### Behavioral Results

Accuracy. The analysis of the proportion of correct responses showed a general tendency to better categorize male rather than female targets (*F*(1, 17) = 9.939, *p* < 0.01, *η*^2^_*p*_ = 0.369) and objectified human rather than doll-like avatars (*F*(1, 17) = 62.438, *p* < 0.001, *η*^2^_*p*_ = 0.786). As expected, target gender and humanity significantly interacted, (*F*(1, 17) = 7.774, *p* < 0.05, *η*^2^_*p*_ = 0.314). Participants were more accurate to recognize doll-like male (M = 84.77, SD = 9.351) compared to doll-like female avatars (M = 79.22, SD = 9.890) (t(17) = −3.104, p < 0.01), while no significant difference emerged between objectified female and objectified male targets (t(17) = −1.045, p = 0.311) (see Fig. SI1 in the Supplemental online Information). This means that participants’ correct recognition was significantly impaired when a doll-like female avatar appeared among a set of objectified female pictures compared to a doll-like male avatar that figured among a set of objectified male pictures.

Reaction time. The required time to give accurate responses was markedly influenced by target gender (*F*(1, 17) = 23.796, *p* < 0.001, *η*^2^_*p*_ = 0.583) and humanity (*F*(1, 17) = 11.248, *p* < 0.01, *η*^2^_*p*_ = 0.398), but was not influenced by the interaction between both variables. Overall, responses were faster for the categorization of male (M = 0.694 s, SD = 0.14) rather than female targets (M = 0.789 s, SD = 0.20) and for objectified humans (M = 0.771 s, SD = 0.17) rather than doll-like avatars (M = 0.772 s, SD = 0.17) (see Fig. SI2 in the Supplemental online Information). It is interesting to note that participants’ responses were impaired towards infrequent rather than frequent stimuli, but that, unlike the accuracy of their responses, they were generally slower at responding to female (both human and avatar) compared to male stimuli. Previous research has shown that pictures of women attract more attention and are looked at for longer periods of time compared to pictures of men^43^. This might have slowed down participants in their reactions towards female rather than male stimuli. This result, however, needs to be interpreted with care as we do not replicate this effect in the following experiments.

### Electrophysiological results

The amplitude of the event-related potential (P300) was strongly influenced by target gender and humanity in all three regions of interest (parietal, occipital and central sites). As expected, the presentation of a female doll-like avatar among objectified female human pictures gave rise to a positive deflection of the P300 that was significantly smaller compared to the presentation of a male doll-like avatar among objectified male pictures. No significant differences were observed between pictures portraying an objectified male and objectified female target (see Figure 1). In all regions, the interaction between target gender and humanity emerged significantly (*F*(1, 17) = 21.786, *p* < 0.001, *η*^2^_*p*_ = 0.562; *F*(1, 17) = 17.791, *p* = 0.001, *η*^2^_*p*_ = 0.511; *F*(1, 17) = 16.573, *p* = 0.001, *η*^2^_*p*_ = 0.494, for occipital, parietal and central sites respectively; see Supporting online Information for the full analysis).

The results support the hypothesis that the P300 is significantly smaller when a female doll-like avatar appears among a set of objectified female pictures compared to when a male doll-like avatar is infrequently presented among a series of objectified male pictures. The amplitude of the P300 in the oddball paradigm depends on two factors: the frequency of the oddball stimulus and the extent to which the infrequent stimuli perceptually differ from the frequent ones. Given that the first factor was kept constant for male and female pictures, these results suggest that objectified female human stimuli are elaborated more similarly to real objects compared to male counterparts. It remains possible, however, that these differences reflect a more general gender effect that is not related to objectified female stimuli per se. To exclude this possibility, we conducted a second experiment with fully-clothed, non-objectified male and female pictures.

## Experiment 2

The procedure of Experiment 2 was similar to the one used in the first experiment. Here, the stimuli portrayed non-objectified (i.e. fully-clothed) male and female targets together with their respective doll-like avatars.

### Results

#### Behavioral Results

Accuracy. Participants’ accuracy was only influenced by target humanity (*F*(1, 17) = 35.679, *p* < 0.001, *η*^2^_*p*_ = 0.677) showing that non-objectified humans (*M* = 95.58, *SD* = 9.95) were categorized more accurately than doll-like avatars (*M* = 83.19, *SD* = 9.63). As expected and in contrast to Experiment 1, no interaction between the gender and the humanity of the targets emerged from the analysis (see Fig. SI3 in the Supplemental online Information).

Reaction time. There were no significant differences on the time spent in categorizing the different stimuli (see Fig. SI4 in the Supplemental online Information).

### Electrophysiological results

The same time windows selected in the first experiment were adopted to extract the mean amplitude in each region of interest. Results revealed no interaction between the gender and the humanity of the target in each region of interest (all *Fs* < 1). Importantly the oddball effect emerged in each ROI with the infrequent doll-like avatars eliciting a more positive wave compared to the frequent non-objectified human targets (*ps* < 0.001). As expected, this effect was not qualified by the gender of the target, although the amplitude of the P300 was overall significantly larger for female compared to male targets (*ps* < 0.05; see Figure 2; see Supporting online Information for a full analysis).

Results of Experiment 2 showed a significant and equally strong oddball effect for both male and female pictures supporting our prediction that the P300 does not differ significantly when a female doll-like avatar appears among a set of non-objectified female pictures compared to when a male doll-like avatar is presented among a series of non-objectified male pictures. In other terms, when the female pictures are fully clothed and not attracting a focus on their bodies they are not objectified and seen equally different from a real object as their male counterparts.

To directly compare the objectified with the non-objectified depictions of male and female targets, an additional analysis was conducted comparing results of both experiments directly. This analysis resulted in a significant interaction between target humanity, target gender and their level of objectification (*F*(1, 34) = 9.125, *p* = 0.005, *η*^2^_*p*_ = 0.21; *F*(1, 34) = 11.252, *p* = 0.002, *η*^2^_*p*_ = 0.249; *F*(1, 34) = 11.526, *p* = 0.002, *η*^2^_*p*_ = 0.253, for occipital, parietal and central sites respectively) demonstrating that only objectified female targets were elaborated more similarly to real objects as compared to all other human targets. As such, not women in general, but only objectified women are seen more similar to objects.

## Experiment 3

In Experiment 1 and 2 the categorization task was always semantically related to the human – object distinction. For this reason, a third experiment was necessary to demonstrate that objectified women are elaborated more similar to objects, even when the human – object dimension is not task relevant. Eliminating any semantic reference allowed us to demonstrate that the “woman object” is not a mere metaphor but that she is perceived more similar to a proper object. In Experiment 3, participants were instructed to categorize the pictures on the basis of a colored contour line that appeared either on the right or the left side of the targets (see Figure 3). The color variable was crossed with the gender of the target resulting in four stimulus blocks. In each block, if the frequent contour line color was green, the infrequent was yellow or vice-versa. The stimuli of Experiment 1 were adapted adding the contour line and, apart from some catch trials (see Methods section for details) the doll-like avatars were always combined with the infrequent color while the human stimuli were paired with the frequent color. It is interesting to note that none of the participants noticed that doll-like avatars appeared among the human stimuli implying that the observed effects occurred outside of participants’ awareness.

### Results

#### Behavioral results

Both the accuracy and reaction time data were not influenced by the humanity or the gender of the targets (see Figs SI5 and SI6 in the Supplemental online Information).

#### Electrophysiological results

The amplitude of the P300 was influenced by both target gender and humanity, only in the occipital area and in a later time window. As expected, the presentation of a female doll-like avatar among objectified female human pictures gave rise to a positive deflection of the P300 that was significantly smaller compared to the presentation of a male doll-like avatar among objectified male pictures (*F*(1, 19) = 10.25, *p* = 0.005, *η*^2^_*p*_ = 0.35). This result confirmed that the male doll-like avatars elicited a more positive activation compared to the female doll-like avatars, *t*(19) = 3.56, *p* = 0.002, *d* = 1.63, while no significant differences occurred between the human objectified male and female targets, *t*(19) = 0.080, *p* = 0.94, *d* = 0.04. Moreover, compared to the objectified, human male stimuli, the male doll-like avatar created a significant positive shift, *t*(19) = −3.63, *p* = 0.002, *d* = −1.67, while no significant difference between the objectified female pictures and their doll-like avatars was observed, *t*(19) = −0.380, *p* = 0.708, *d* = −0.17 (see Figure 3; see Supporting online Information for a full analysis).

## Discussion

To what extent does a “she” become an “it” when objectified? Is the perception of women as objects a mere metaphor or does the objectification of women convey true similarities with real objects? To answer this question, the present research directly assesses participants’ neural patterns when elaborating objectified women and real comparable objects. Results show that objectified women are perceived more similar to real objects. Experiment 1 demonstrated this result comparing objectified female with objectified male targets, while the results of Experiment 2 confirmed that this effect is confined to objectified depictions of women. Non-objectified female and male human targets were equally and clearly differentiated from doll-like objects. These results similarly reflected in participants’ behavioral responses showing that doll-like female objects were significantly less well recognized when they appeared among a set of objectified female pictures compared to objectified and non-objectified doll-like male and non-objectified doll-like female objects that appeared among their human counterparts. Results of Experiment 3 allowed us to conclude that even when no semantic reference to the human-object divide is provided, objectified women are still perceived as more similar to objects. As a matter of fact, in the latter case no oddball effect was observed meaning that people did not elaborate the female human and female doll-like objects differently in any way. It is important to acknowledge that this effect was only found in a more posterior region and in a shorter post-stimulus time window compared to the former experiments. It is generally known that stimulus and task requirements change the latency of the P300^41^ and the task of Experiment 3 showed to be slightly more difficult (*M*_*accuracy*_ = 82%) compared to the former ones (*M*_*accuracy*_ = 89.6% and 89.3% for Experiment 1 and 2 respectively). Moreover, the P300 component is sensitive to task relevance. Therefore, disconnecting the rules of the current task based on color from the hypothesis inevitably diminished the strength of the interaction effect to a single ROI. Admittedly, it remains currently unclear why this effect should be mainly localized in the occipital area.

In our studies we used stimuli that may be associated with variations in sensory parameters (such as form, luminance or contrast). Previous research (e.g.^44^) has shown that such variations have a direct effect on the early ERP responses (i.e., within 200 ms post-stimulus onset, such as the P1 and N1). However, the present results did not indicate differences between the current stimuli in the early time-window. This implies that these bottom-up perceptual processes did not play a major role in our findings. This result is in line with the results of our pre-test in which the doll-like avatars that were used in all experiments were judged as equally object-like, regardless of their gender or the way they were dressed, and were kept perceptually as similar as possible to their human originals. Moreover, finding the expected interaction only in a later time-window allows us to conclude that top-down processes played a central role in our studies. Finally, it is important to highlight that the overall pattern of results was equally strong for male and female participants suggesting that participants of both genders erroneously perceive objectified women more similar to true objects than objectified men to the same extent. Taken together these data support the notion that when a woman is objectified, because of her revealing clothing or suggestive posture^45^, she will be perceived as similar to a real object.

These results have important implications. First, perceiving women as objects might justify treatments that are typically observed in our interaction with objects, like ownership and violation^46^. Secondly, the finding that female doll-like avatars are less clearly differentiated from real women might imply that the recurrent sexualization of women in media or video games^6^ might have stronger effects in real life compared to hyper-masculine virtual representations. While no research has tested this idea directly, indirect evidence was provided showing that men who were exposed to sex-typed video game characters, compared to professional men and women, increased their tolerance for a real-life instance of sexual harassment^47^ and increased their likelihood to sexually harass a female target when playing a sexually explicit video game^48^. Third, the current paradigm might be adopted to measure objectification and dehumanization processes in other contexts as well (i.e., medical objectification or race- or nation-based dehumanization). With the only use of trait, associative or metaphorical measures, it remains difficult to claim that objectified or dehumanized targets change in essence rather than just being stereotyped as less intelligent or less evolved^49^. Adopting the current paradigm that directly measures whether human and non-human entities are perceived differently might provide evidence of processes of dehumanization beyond the metaphor.

## Methods

### Experiment 1

#### Participants

Sample size was determined on the basis of a power analyses. The effect sizes (*η*_*p*_^2^ ranging from 0.504 to 0.709) that were reported in previous work using^40^ the oddball paradigm with pictorial stimuli in a similar within-participants design, were rather large. Therefore, it seemed reasonable to expect half the effect size they reported for the current studies. A power analysis (PANGEA^50^) suggested that a sample of 16 participants would be sufficient to detect an interaction effect with a power of 0.825. Therefore, we decided to collect around 20–25 participants in each study. In Experiment 1, a total of twenty-three healthy volunteers participated in the experiment. All participants had normal or corrected to normal vision and reported no history of neurological impairment. Only participants who indicated to be heterosexual were retained in the sample, resulting in the exclusion of three homosexual participants. Two further participants were excluded from the analyses because of a very poor signal-to-noise ratio caused by an excessive rate of EEG artifacts (exceeding 25%). All analyses were carried out on the data of 18 participants (8 female; *M*_*age*_ = 20.66, *SD* = 1.29). The methods of all studies were carried out in accordance with the experimental protocol (2016-004) that was approved by the “Comitato Etico per la sperimentazione con l’essere umano”. Informed consent was obtained from all participants at the beginning of the experiment.

#### Apparatus

Testing occurred individually in a sound attenuating, dimly lit and electrically shielded booth. Participants were seated at a distance of 80 cm from a 23.6 inch color monitor (1920x1080, 120 Hz) placed in front of the participant. Stimuli were generated by MATLAB Psychotoolbox.

#### Stimuli and procedure

There were 82 stimuli, 42 representing females (21 objectified female and 21 resembling female doll-like avatar targets) and 40 males (20 objectified male and 21 resembling male doll-like avatar targets; see Figure 1). The dimension of all pictures was 5.35° × 7.64°. The stimuli were presented 2.67° under the center of the monitor and on a uniformly gray background at the center of the screen. The fixation cross was located 1.91° above the center of the screen.

We used an oddball paradigm that involved the presence of an infrequent stimulus (doll-like avatar) within a sequence of frequent stimuli (objectified human targets)^41^. Participants were required to perform a categorization task, in which they had to indicate as accurately and as fast as possible whether each picture portrayed either a doll-like avatar or a human target, by means of a key press. The experiment was divided in four blocks with a randomized order between subjects: two blocks contained human and doll-like female targets, while the remaining two blocks consisted of male human and doll-like targets. Each block included 250 stimuli (80% frequent stimuli and 20% infrequent stimuli). In this way, the presentation of sequences of repetitive stimuli of objectified human targets were infrequently interrupted by a deviant stimulus representing doll-like avatar targets, with the constraint that at least two frequent stimuli would be presented before an infrequent one. Each trial began with a 1500 ms presentation fixation cross (+) 1.91° above the center of the screen. Afterwards, the stimulus remained on the screen until participants made their judgment.

### Experiment 2

#### Participants

Twenty-two healthy volunteers took part in Experiment 2. All participants had normal or corrected to normal vision and reported no history of neurological impairment. Data from one participant, who indicated to be bisexual, were discarded from further analyses. In addition, two participants were excluded because their EEG signal was contaminated by many artifacts (exceeding 25%). As a result, 18 participants (8 female, *M*_*age*_ = 22.97, *SD* = 2.24) were retained for further analysis.

#### Stimuli and procedure

The apparatus was identical to that used in Experiment 1. The 82 stimuli now represented non-objectified male and female targets (i.e. fully-clothed individuals) and their equivalent male and female doll-like avatars. In the non-objectified stimuli less skin was visible, thus making the task more difficult with respect to Experiment 1. For this reason, the task was made comparably difficult to Experiment 1 increasing the stimulus size (8.02° × 11.46° from stimulus center). The center of all pictures was located 4° under the midpoint of the screen, while the fixation cross appeared 2.29° above the center of the monitor. The procedure was the same as the one we used in Experiment 1.

### Experiment 3

#### Participants

Twenty-nine participants were enrolled either for course credits or paid 10€ for their participation. All participants had normal or corrected vision and no history of neurological disease. Data from 9 participants were discarded from further analyses (5 participants indicated to be non-heterosexual, 3 participants made more than 25% errors, and 1 participant was already familiar with the target pictures of the experiment). The final sample consisted of 20 participants (10 male; *M*_*age*_ = 21.2, *SD* = 2.08).

#### Stimuli and procedure

The same pictures as those in Experiment 1 were adapted adding a yellow (227-40-30 RGB) or green (112-235-44 RGB) contour on the right or on the left side of the target body. The dimension of the contour was 0,3 mm, and the brightness of both colors was equalized. The background color of each picture was the same as that of the screen, in this way the pictures appeared without any frame. Here, the frequent and infrequent stimuli were differentiated on the basis of the color of the contour of the pictures and were categorized by means of a key press. In most cases, the infrequent color was paired with the doll-like avatars, while the frequent color was applied to the human targets. Four experimental blocks were created that differed in terms of the gender of the target and the frequent color (yellow or green). Each block consisted of 250 regular stimuli (80% frequent target and 20% infrequent target) and 25 catch trials. The catch trials were created to avoid a learning effect and the possibility to categorize the stimuli using a double categorization criterion. In these trials, the frequent color was matched with the doll-like avatars (in 20 trials), while the infrequent color with the human targets (in 5 trials). The catch trials were excluded from all analyses.

#### EEG acquisition

In all experiments, EEG was recorded from the scalp with 25 electrodes and a left earlobe electrode, with a right earlobe reference (bandpass filter: 0.01–200 Hz; A/D rate: 1000 Hz). Electrode impedance was maintained below 5 KΩ.

Data analysis were conducted using the EEGLAB^51^ and ERPLAB toolbox^52^. Raw data were digitally filtered with a bandpass filter of 0.1–40 Hz. The EEG data were re-referenced offline to the average of the right and left earlobe electrodes. The horizontal electrooculogram (HEOG) was recorded from two electrodes placed on the outer canthi of both eyes. The signal was segmented in 900ms-long epochs that began 100 ms prior to trial onset. Baseline correction was applied using the mean activity during the 100 ms pre-stimulus interval. Trials with horizontal eye movements (HEOG exceeding ± 30 µV) or other movement artifacts (any channel exceeding ± 70 µV) were rejected. The mean number of retained trials for each participant was 85%. ERP averages for correct responses were computed for each condition. ERPs were tested statistically after data were averaged across channels in three separated regions of interest (ROI): central (electrodes Cz, C3, C4); parietal (electrodes Pz, P3, P4) and occipital (electrodes Oz, O1 and O2).

### Data analysis

All analyses were conducted using SPSS software. Behavioral responses were assessed for each participant by calculating the mean reaction times for correct trials and the mean percentage of correct responses. A two-way within-participant ANOVA testing the impact of target gender (male vs. female) and humanity (human vs. doll-like avatars) was conducted separately for response times and accuracy. Given that participants’ gender never showed any main or interaction effects with the other variables of interest, the variable was excluded from the analyses. Therefore, all reported results hold for both male and female participants.

To quantify the time intervals for the P3 for each ROI we used a data-driven approach. First, we conducted multiple 2 (Target gender: male vs. female) ×2 (Humanity: human vs. doll-like avatars) within-participant ANOVAs on 20 ms time windows starting from stimulus onset and selected the time windows for which the interaction between target gender and humanity remained significant across at least 5 consecutive windows (i.e., 100 ms) (see^53^ for the use of a similar approach). On the basis of these results, the main ANOVAs were conducted separately for each ROI in the following time windows: central 400–580 ms, parietal 360–600 ms and occipital region 360–600 ms. All raw data are made available in a public repository (https://osf.io/ejhmf/?view_only=734f9ae8f6884802b13cf461a535f60d).

## Supplementary information

Supplemental online Information
