# Supplementary material for: Assessing neural responses towards objectified human targets and objects to identify processes of sexual objectification that go beyond the metaphor
Source: Sci Rep. 2019 Apr 30;9:6699. doi: 10.1038/s41598-019-42928-x (PMC6491438; doi:10.1038/s41598-019-42928-x)
Supplement: Supplementary file 1 — Supplemental online Information [file 41598_2019_42928_MOESM1_ESM.docx]

Supplemental online Information

Assessing neural responses towards objectified human targets and objects to identify processes of sexual objectification that go beyond the metaphor

Jeroen Vaes, Giulia Cristoforetti, Daniela Ruzzante, Carlotta Cogoni, & Veronica Mazza

**Pre-test supplemental analyses:**

To assess the validity of our set of stimuli a pre-test was conducted. Apart from recognizing the pictures as representing a human or an object (see main manuscript for this analysis), participants (*N*=22, 12 female) were asked to rate the beauty, sexiness and in the case of the human images the level of objectification on a 7-point Likert scale. Each dimension was separately analyzed by means of a 2 (Target: male vs. female) X 2 (Humanization: human vs. doll-like avatar) X 2 (Objectification: Objectified vs. Non-objectified) X 2 (Participants’ gender: male vs. female) mixed ANOVA in which only the last variable was manipulated between participants.

Results on the beauty dimension showed that images of female targets (*M* = 3.62, *SD* = .75 were rated as more beautiful than the images representing male targets (*M* = 2.91, *SD* = .67) *F*(1, 20)=33.07, *p*<.001, *η^2^_p_*=.62. Human images (*M* = 4.42, *SD* = .66) were also rated more beautiful than the doll-like avatars (*M* = 2.11, *SD* = .88), *F*(1, 20)=160.17, *p*<.001, *η^2^_p_*=.89. No other effects were statistically significant (all ps>.07).

Results on the sexiness dimension indicated that female images (*M* = 3.35, *SD* = .70) were rated sexier than the male ones (*M* = 2.62, *SD* = .67), *F*(1, 20)=35.20, *p*<.001, *η^2^_p_*=.64. Human images (*M* = 4.11, *SD* = .83) were rated as sexier than the doll-like avatars (*M* = 1.87, *SD* = .74), *F*(1, 20)=113.35, *p*<.001, *η^2^_p_*=.85. In addition, Target significantly interacted with Humanization *F*(1, 20)=6.67, *p*=.02, *η^2^_p_*=.25, indicating that even though the female image was always rated sexier than the male image, this effect was stronger for the human compared to the doll-like avatars (*M* = 4.60, *SD* = .87 and *M* = 3.61, *SD* = 1.05, *p*<.001 for the female human and male human images respectively and *M* = 2.10, *SD* = .94 and *M* = 1.63, *SD* = .58, *p*<.001 for the female object and male object images respectively). This interaction was further qualified by Participants’ gender, *F*(1, 20)=4.65, *p*=.04, *η^2^_p_*=.25: female images were always rated as sexier than male images, but while male participants displayed this difference stronger in human as compared to object stimuli, female participants displayed the opposite pattern. No other effect was statistically significant (all ps>.11).

**Figures for the behavioral results of Experiment 1.**


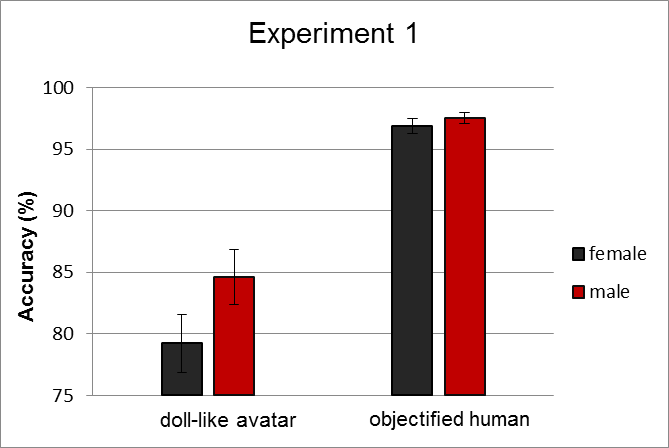


*

***Fig. SI 1****: Mean proportion of correct responses in categorizing female and male targets as a function of their humanity (avatar vs. human. Error bars represent standard errors. * represents a significant difference p<.05.*

*Note.* Due to a programming error, two male stimuli, one human and one avatar, were coded wrongly. We decided to exclude these trials from any further analyses. As a result, the male accuracy index is calculated on a total of 498, instead of 500 observations.


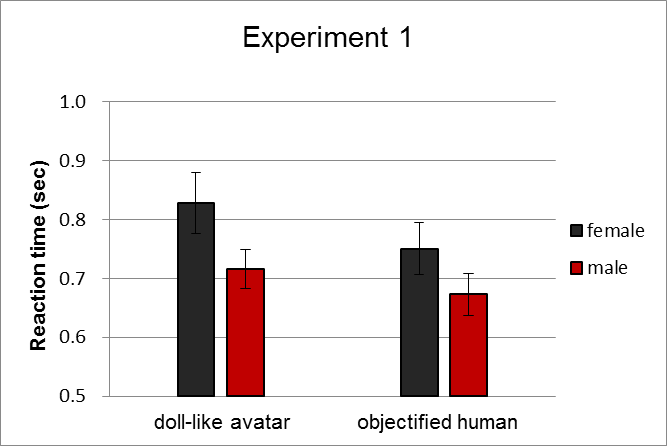


**Fig. SI 2**: Mean reaction times as a function of target gender and humanity (avatar vs. human). Error bars represent standard errors.

**All Electrophysiological Results of Experiment 1.**

In each Region Of Interest (ROI), a 2 (male/female target) x 2 (doll-like avatar/objectified human) ANOVA was carried out with all factors manipulated within participants. For the occipital site (360-600 ms post-stimulus) the analyses showed a main effect of gender (*F*_(1,17)_=13.15, *p*=.002, *η^2^_p_*=.44) and humanity (*F*_(1,17)_=93.93, *p*<.001, *η^2^_p_* = .85). Male targets elicited a more positive P300 compared to female targets; and doll-like avatars elicited a more pronounced response compared to human targets. Further, the expected interaction between gender and humanity emerged significantly (*F*_(1,17)_=21.79, *p*<.001, *η^2^_p_*=.56). As expected, the presentation of a male doll-like avatar among objectified male pictures gave rise to a more positive deflection of the P300 than the presentation of a female doll-like avatar among objectified female pictures (t_(17)_=4.79, p<.001). The difference between pictures portraying objectified male and objectified female human targets was not significant (t_(17)_=1.21, p=.243).

In the parietal region (360-600 ms post-stimulus), the ANOVA revealed similar effects to the ones in the occipital region. The analysis showed a main effect of gender (*F*_(1,17)_=6.64, *p*=.020, *η^2^_p_*=.28) and humanity (*F*_(1,17)_=140.23, *p*<.001, *η^2^_p_*=.89) and there was a two-way significant interaction between gender and humanity (*F*_(1,17)_= 17.79, *p*=.001, *η^2^_p_*=.51). Similarly, the P300 elicited by male doll-like avatars was more positive than the one that was triggered by the female doll-like avatars (t_(17)_=3.24, p=.005). No significant difference was observed between the objectified male and female human targets (t_(17)_=0.67, p=.51).

For the central site, an ANOVA was computed in the time window: 400-580 ms post-stimulus. We obtained the same effects and interaction as in the occipital and parietal region. There was a main effect of gender ((*F*_(1,17)_=7.14, *p*=.016, *η^2^_p_* =.29 ) and humanity (*F*_(1,17)_= 96.38, *p*<.001, *η^2^_p_* =.85) and also the interaction emerged significantly (*F*_(1,17)_=16.57, *p*=.001, *η^2^_p_*=.49). There was a significant difference between female doll-like avatars and male doll-like avatars (t_(17)_=3.21, p=.005; see **Table S1)**.

The results support our hypothesis in all ROIs; the P300 is significantly smaller when a female doll-like avatar appears among a set of objectified female pictures compared to when a male doll-like avatar is infrequently presented among a series of objectified male pictures.

***Table S1.*** *Experiment 1. Means (standard errors) µV for the P300 in the three regions of interest as a function of gender and humanity*

|  |  | Male targets | | Female targets | |
| --- | --- | --- | --- | --- | --- |
|  |  | Doll-like avatar | Objectified human | Doll-like avatar | Objectified human |
| Occipital region | 360 – 600 ms | 27.59  (3.83) | 10.44  (2.88) | 20.32  (2.99) | 9.04  (2.43) |
| Parietal region | 360 – 600 ms | 23.38  (3.57) | 4.97  (2.38) | 16.36  (3.60) | 4.35  (2.59) |
| Central region | 400 – 580 ms | 20.79  (4.91) | 1.45  (3.52) | 11.61  (5.19) | .16  (3.85) |
|  |  |  |  |  |  |

**Figures for the behavioral results of Experiment 2**


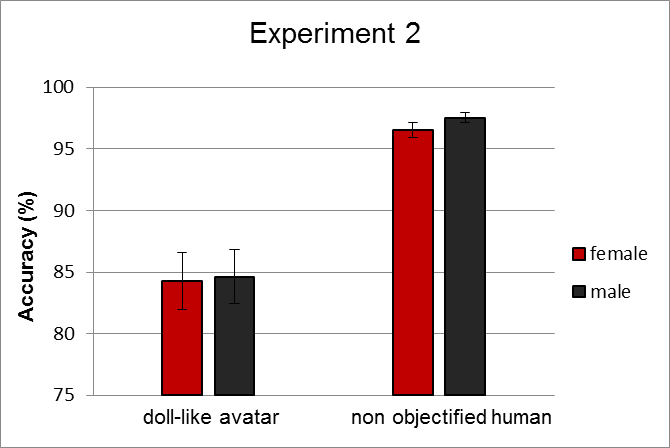


***Fig. SI 3****: Mean proportion of correct responses in categorizing female and male targets as a function of their humanity (avatar vs. human. Error bars represent standard errors.*


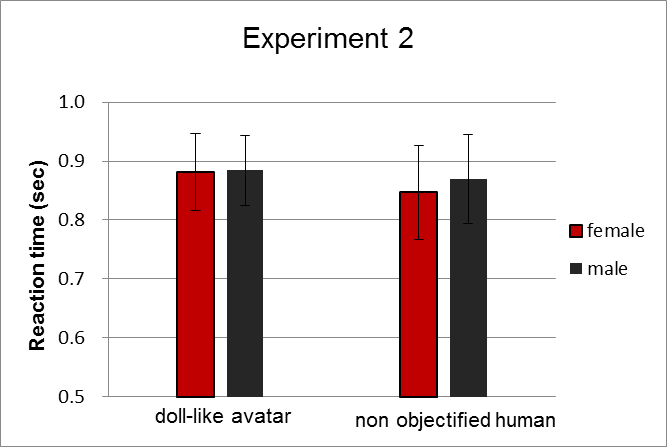


**Fig. SI 4**: Mean reaction times as a function of target gender and humanity (avatar vs. human). Error bars represent standard errors.

**All Electrophysiological Results of Experiment 2**

For the occipital, parietal and central ROIs, we computed separate two-way ANOVAs, with gender (male/female) and humanity (non-objectified human/doll-like avatar) as factors. Mean amplitude values were extracted in each ROI for the same time windows used in Experiment 1.

The analysis in the occipital region (360-600ms post-stimulus) showed both a main effect of gender (*F*_(1,17)_=7.07, *p*=.016, *η^2^_p_*=.29) and humanity (*F*_(1,17)_=34.08, *p*<.001, *η^2^_p_*=.67). The interaction did not emerge (*F*_(1,17)_= .27, *p*=.61, *η^2^_p_* =.016). For the parietal region (360-600ms post-stimulus), the analysis showed the significant effects of gender (*F*_(1,17)_=15.95, *p*=.001, *η^2^_p_*=.48) and humanity (*F*_(1,17)_=28.59, *p*<.001,*η^2^_p_*=.63), but no significant interaction (*F*_(1,17)_= .38, *p*=.547, *η^2^_p_*=.022). In the central sites (400-580ms post-stimulus), there was a main effect of gender *F*_(1,17)_=14.94, *p*=.001, *η^2^_p_*=.47) and humanity (*F*_(1,17)_=20.12, *p*<.001, *η^2^_p_*=.54), but no significant interaction effect (*F*_(1,17)_=1.005, *p*=.330, *η^2^_p_*=.05). Taken together, these results support our prediction that the P300 does not differ significantly when a female doll-like avatar appears among a set of non-objectified female pictures compared to when a male doll-like avatar is presented among a series of non-objectified male pictures. (**Table S2.)**

***Table S2:*** *Experiment 2. Means (standard errors) µV for the P300 in the three regions of interest as a function of gender and humanity*

|  |  | Male targets | | Female targets | |
| --- | --- | --- | --- | --- | --- |
|  |  | Doll-like avatar | Non-objectified human | Doll-like avatar | Non-objectified human |
| Occipital region | 360 – 600 ms | 23.63  (3.64) | 12.58  (2.76) | 25.39  (3.55) | 14.96  (2.50) |
| Parietal region | 360 – 600 ms | 14.35  (3.82) | 3.90  (2.39) | 18.90  (4.14) | 7.48  (2.69) |
| Central region | 400 – 580 ms | 8.00  (4.56) | -1.76  (2.77) | 14.14  (5.25) | 2.46  (3.50) |
|  |  |  |  |  |  |

Figures for the behavioral results of Experiment 3

**
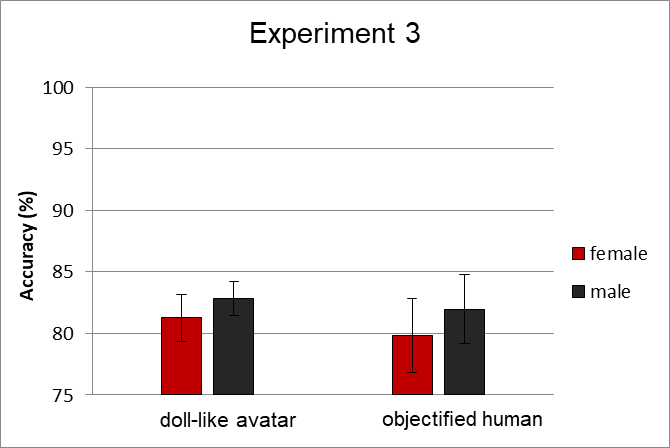
**

**Fig. SI 5**: Mean proportion of correct responses in categorizing female and male targets as a function of their humanity (avatar vs. human. Error bars represent standard errors.


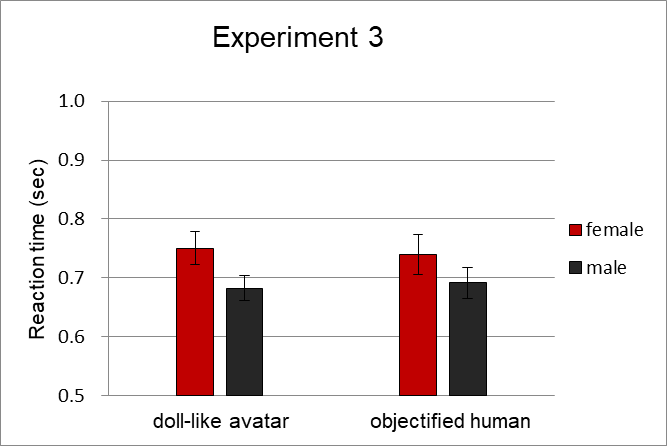


***Fig. SI 6****: Mean reaction times as a function of target gender and humanity (avatar vs. human). Error bars represent standard errors.*

**All Electrophysiological Results of Experiment 3**

We compute the same two-way ANOVAs gender (male/female) x humanity (objectified human/doll-like avatar) in the three regions of interest separately. Due to a different nature of the task, in the third experiment we extracted the mean amplitude in a different time window. Specifically, we analysed the 440-540ms post-stimulus time window in each ROI. The hypothesis was confirmed only in the occipital site. A main effect of humanity *(F(1, 19)=9.20, p=.007, η^2^_p_=.32)* emerged, while gender was marginally significant *(F(1, 19)=4.39, p=.051, η^2^_p_=.18)*. Specifically, the doll-like avatars elicited a more positive P300 compared to the objectified human targets and the male targets elicited a stronger P300 compared to female targets. In line with our hypothesis, the interaction between gender and humanity was significant *(F(1, 19)=10.25, p=.005, η^2^_p_=.35)*. This result confirmed that the male doll-like avatars elicited a more positive activation compared to the female doll-like avatars *(t_(19)_= 3.56, p=.002)*, while no significant differences occurred between the human objectified male and female targets *(t_(19)_=-.080, p=.93)*. Moreover, compared to the objectified, human male stimuli, the male doll-like avatar created a significant positive shift (t_(19)_=-3.63, p=.002), while no significant difference between the objectified female pictures and their doll-like avatars was observed (t_(19)_=-.380, p=.708; see **Table** **S3**).

In the same time window, analyses in the parietal sites showed a main effect of gender *(F(1, 19)=24.86, p<.001, η^2^_p_=.57)* and humanity *(F(1, 19)=9.36, p=.006, η^2^_p_=.33)*. The male doll-like avatars elicited a more positive activation compared to the female doll-like avatars and overall the doll-like avatars showed a stronger positive activation compared to the human stimuli. The interaction between gender and humanity did not emerge *(F(1, 19)=2.22, p=.15, η^2^_p_=.105)*. In the central site, the analyses showed a significant gender effect *(F(1, 19)=28.55, p<.001, η^2^_p_=.60)*, while humanity had no effect *(F(1, 19)=3.25, p=.087, η^2^_p_=.14)*. Again, male targets showed a stronger positive activation compared to the female targets. No significant interaction was observed between gender and humanity *(F(1, 19)=.53, p=.47, η^2^_p_=.027)*.

These results support the hypothesis that the P300 was more positive during the presentation of the male doll-like avatars among male objectified targets than the presentation of female doll-like avatar among objectified female targets. This activation was only observed in a more posterior site. Interestingly, the difference between the female doll-like avatars and the objectified human targets disappeared completely suggesting that the objectified women were not differentiated anymore from the real objects (see **Table S3).**

***Table S3:*** *Experiment 3. Means (standard errors) µV for the P300 in the three regions of interest as a function of gender and humanity*

|  |  | Male targets | | Female targets | |
| --- | --- | --- | --- | --- | --- |
|  |  | Doll-like avatar | Objectified human | Doll-like avatar | Objectified human |
| Occipital region | 440 – 540 ms | 8.37  (2.67) | 5.18  (2.48) | 5.31  (2.66) | 5.108  (2.708) |
| Parietal region | 440 – 540 ms | 6.73  (1.77) | 3.39  (1.77) | 1.53  (2.13) | .645  (1.58) |
| Central region | 440 – 540 ms | 6.10  (2.37) | 3.34  (2.38) | .58  (2.80) | -.257  (1.85) |
|  |  |  |  |  |  |
